# Identification of the minimum region of flatfish myostatin propeptide (Pep45-65) for myostatin inhibition and its potential to enhance muscle growth and performance in animals

**DOI:** 10.1371/journal.pone.0215298

**Published:** 2019-04-18

**Authors:** Jeong Hwan Kim, Jeong Han Kim, Lisa Andriani Sutikno, Sang Beum Lee, Deuk-Hee Jin, Yong-Ki Hong, Yong Soo Kim, Hyung-Joo Jin

**Affiliations:** 1 Department of Marine Molecular Bioscience, Gangneung-Wonju National University, Gangneung-si, Ganwon-do, Korea; 2 Department of Biotechnology, Pukyong National University, Namgu, Busan, Korea; 3 Department of Human Nurtrition, Food and Animal Sciences, University of Hawaii, Honolulu, Hawaii, United States of America; University of Tennessee Health Science Center College of Graduate Health Sciences, UNITED STATES

## Abstract

Myostatin (MSTN) negatively regulates skeletal muscle growth, and its activity is inhibited by the binding of MSTN propeptide (MSTNpro), the N-terminal domain of proMSTN that is proteolytically cleaved from the proMSTN. Partial sequences from the N-terminal side of MSTNpro have shown to be sufficient to inhibit MSTN activity. In this study, to determine the minimum size of flatfish MSTNpro for MSTN inhibition, various truncated forms of flatfish MSTNpro with N-terminal maltose binding protein (MBP) fusion were expressed in *E*. *coli* and purified. MSTNpro regions consisting of residues 45–68, -69, and -70 with MBP fusion suppressed MSTN activity with a potency comparable to that of full-sequence flatfish MSTNpro in a pGL3-(CAGA)_12_-luciferase reporter assay. Even though the MSTN-inhibitory potency was about 1,000-fold lower, the flatfish MSTNpro region containing residues 45–65 (MBP-Pro45-65) showed MSTN-inhibitory capacity but not the MBP-Pro45-64, indicating that the region 45–65 is the minimum domain required for MSTN binding and suppression of its activity. To examine the *in vivo* effect of MBP-fused, truncated flatfish MSTNpro, MBP-Pro45-70-His6 (20 mg/kg body wt) was subcutaneously injected 5 times for 14 days in mice. Body wt gain and bone mass were not affected by the administration. Grip strength and swimming time were significantly enhanced at 7 d after the administration. At 14 d, the effect on grip strength disappeared, and the extent of the effect on swimming time significantly diminished. The presence of antibody against MBP-Pro45-70-His6 was observed at both 7 and 14 d after the administration with the titer value at 14 d being much greater than that at 7 d, suggesting that antibodies against MBP-Pro45-70-His6 neutralized the MSTN-inhibitory effect of MBP-Pro45-70-His6. We, thus, examined the MSTN-inhibitory capacity and *in vivo* effect of flatfish MSTNpro region 45–65 peptide (Pep45-65-NH2), which was predicted to have no immunogenicity in silico analysis. Pep45-65-NH2 suppressed MSTN activity with a potency similar to that of MBP-Pro45-65 but did not suppress GDF11, or activin A. Pep45-65-NH2 blocked MSTN-induced Smad2 phosphorylation in HepG2 cells. The administration of Pep45-65 (20 mg/kg body wt, 5 times for 2 weeks) increased the body wt gain with a greater gain at 14 d than at 7 d and muscle wt. Grip strength and swimming time were also significantly enhanced by the administration. Antibody titer against Pep45-65 was not detected. In conclusion, current results indicate that MSTN-inhibitory proteins with heterologous fusion partner may not be effective in suppressing MSTN activity *in vivo* due to an immune response against the proteins. Current results also show that the region of flatfish MSTNpro consisting of 45–65 (Pep45-65) can suppress mouse MSTN activity and increase muscle mass and function without invoking an immune response, implying that Pep45-65 would be a potential agent to enhance skeletal muscle growth and function in animals or to treat muscle atrophy caused by various clinical conditions.

## Introduction

Myostatin (MSTN), a member of the TGF-β superfamily, is a negative regulator of skeletal muscle development and growth. Knockout of the MSTN gene resulted in a dramatic increase in skeletal muscle mass in mice with little effect on other organs [1], while systemic overexpression of MSTN by transgenesis or selective gene transfer in skeletal muscle induced profound muscle atrophy in mice [2–4]. Owing to the potent inhibitory role of MSTN on muscle growth, there has been much interest in MSTN-blocking as a strategy to treat muscle atrophy caused by chronic diseases such as cancer, kidney failure, obstructive pulmonary disease, cardiomyopathy, liver cirrhosis, and age-associated sarcopenia [5–7]. Approaches like the administration of MSTN-blocking proteins or peptides [8–14], delivery of MSTN-blocking genes using an adeno-associated viral vector [15–17], and micro or short hairpin RNA inhibition of MSTN [18, 19] have been used to block MSTN activity. Building on the results in lab animals, some of the MSTN-inhibitors, such as antibodies against MSTN, MSTN peptibody and soluble form of ActRIIB, have been applied in clinical trials [20–23].

MSTN propeptide (MSTNpro), the N-terminal domain of proMSTN that is proteolytically cleaved from the proMSTN, suppresses MSTN activity by the complex formation in a latent/inactive state [4, 24, 25]. Members of the bone morphogenetic proteins-1/tolloid (BMP-1/TLD) of metalloproteinases specifically cleaves MSTNpro, leading to activation of MSTN by separating it from the MSTNpro/MSTN latent complex [26, 27]. A dramatic increase in skeletal muscle mass was observed in transgenic mice overexpressing MSTNpro [28–30]. MSTNpro abundance *in vivo* by various approaches, including plasmid-mediated delivery [31], adeno-associated virus vector [17], and protein administration [9, 11, 27, 32], also resulted in an increase in muscle mass, enhancement of muscle repair or amelioration of dystrophic pathophysiology in mice. These results together indicate that MSTNpro would be a potential agent to treat muscle atrophy caused by various chronic diseases and conditions.

Studies have shown that mouse MSTNpro region containing amino acid residues within 42–115 is effective in suppressing MSTN activity *in vitro* [12, 13, 33, 34]. Furthermore, intramuscular injection of the truncated MSTNpro peptides increased muscle mass in Duchenne and caveolin 3-deficient limb-girdle muscular dystrophy model mice [12, 13]. In our study, maltose binding protein (MBP)-fused pig MSTNpro region containing residues 42–175 exhibited full MSTN-inhibitory potency [35]. Similarly, flatfish MSTNpro region containing residues 45–80 showed MSTN-inhibitory potency comparable to that of full sequence mouse MSTNpro [36], indicating that MBP fused, truncated MSTNpro peptides would be potential agents to treat muscle wasting conditions. According to Takayama et al. [13], mouse MSTNpro region containing residues 43–67 was effective in suppressing the MSTN activity. It was thus, hypothesized that the minimum region of flatfish MSTNpro for MSTN suppression would potentially be smaller than the region containing residues 45–80. The objective of this study was to determine the minimum size of flatfish MSTNpro for MSTN inhibition and examine its effects on muscle growth and performance in mice.

## Materials and methods

### Construction of expression vectors

To construct various truncated forms of flatfish MSTN1 prodomain (MSTNpro), we performed site-directed mutagenesis of an expression plasmid (*pMAL-c5x-Pro45-80*) using the Q5 site-directed mutagenesis kit (NEB, USA). The *pMAL-c5x-Pro45-80* contains the flatfish MSTNpro cDNA fragment covering the amino acid sequence from 45 to 80 [36]. For site-directed mutagenesis, the primers (S1 Table) were designed using NEBaseChanger tool (NEB, MA, USA). PCR products were treated with KLD enzymes (NEB, MA, USA) to remove the phosphorylated DNA terminus and methylated template DNA. After KLD reaction, the reactants were transformed into DH5a competent cells (Solgent, Seoul, Korea) by the heat shock method and spread on Luria-Bertani (LB) agar plate (1.0% tryptone, 0.5% yeast extract, 1.0% NaCl, 1.0% agar) containing 100 μg/mL ampicillin. Selected colonies were inoculated in 8 ml of LB medium containing 100 μg/mL ampicillin and incubated at 37 °C for overnight. Plasmids were extracted using a plasmid extraction kit (Solgent, Seoul, Korea) to confirm correct insertion by sequencing analysis (Solgent, Seoul, Korea).

### Expression and purification of MBP-fused truncated MSTNpros

The expression plasmids were transformed into *E*. *coli* strain K12TB1 (NEB, USA) separately by the heat-shock method. Selected colonies were inoculated in 8 ml of LB medium containing 100 μg/mL ampicillin plus 10 μg/mL streptomycin and incubated at 37 °C for overnight. For the expression of recombinant proteins, the 8 mL overnight-cultures were inoculated in 500 ml of fresh LB medium containing 100 μg/mL ampicillin plus 10 μg/mL streptomycin and incubated at 37 °C. When OD_600_ reached 0.3~0.4, IPTG was added to the cultures to a final concentration of 0.3 mM, and the cultures were incubated at 37 °C for 3 h. Cells were harvested by centrifugation at 4,000 rpm, 4 °C for 20 min. Pellets were resuspended in 20 mL of column buffer (20 mM Tris-HCl, pH 8.0, 200 mM NaCl, 1 mM EDTA) containing 1 mM PMSF and were disrupted by sonication for 10 min in an ice water bath. The lysate was centrifuged at 12,000 rpm for 20 min at 4 °C, and the supernatant was collected. MBP-fused truncated MSTNpro proteins were affinity-purified using amylose resin (NEB, MA, USA) as described previously (Lee et al., 2012). Protein concentration was determined by the BCA assay (Thermo Scientific, MA, USA) using BSA as a standard.

### Expression and two-step affinity purification of MBP-Pro45-70-His6

A His6 tag was inserted at the C-terminus of pMALc5x-Pro45-70 by site-directed mutagenesis with primer sets in S1 Table. Sequencing analysis confirmed the insertion of the His6 tag. Protein expression and preparation of cell lysates were carried out as described previously. *E*. *coli* lysate was centrifuged at 12,000 rpm for 20 min at 4°C, and the supernatant was affinity-purified using Ni-NTA agarose (Qiagen, MD, USA). After loading, the column was washed with binding buffer and eluted with elution buffer (20 mM Tris-HCl, pH 8.0, 500 mM NaCl, 40 mM imidazole). Elution fractions were subjected to an amylose resin affinity column. Eluted fractions containing the MBP-Pro45-70-His6 were pooled and dialyzed extensively against ultra-pure water using dialysis tubing (Spectrum Inc, Seoul, Korea). Protein concentration was determined by the BCA assay (Thermo Scientific. MA, USA), and then was dried by a speed vacuum concentrator and stored at -20°C until use.

### SDS-PAGE

SDS-PAGE (10% polyacrylamide gel) was performed according to the method of Laemmli [37]. Samples were mixed with loading buffer in the presence of 1.5% β-mercaptoethanol and incubated at 95 °C for 5 min before loading on the gel. Bands were stained with Coomassie brilliant blue solution.

### pGL3-(CAGA)_12_ luciferase assay

The anti-MSTN activity was measured using a pGL3-(CAGA)_12_-luciferase reporter assay in HEK293 cells stably expressing (CAGA)_12_-luciferase gene construct [38]. HEK293 cells were seeded on 96 well plates at 2×10^4^ cells//well and grown in DMEM containing 10% FBS, 1% penicillin/streptomycin and 1% geneticin at 37 °C with 5% CO_2_ for 24hr. After 24 hr, the medium was replaced with 100 μL serum-free DMEM. Then 1 nM MSTN, Activin A or Growth and Differentiation factor 11 (GDF11) (R&D systems, MN, USA) plus various concentrations of truncated flatfish MSTNpro were added to each well and incubated for 24 h. After incubation, luciferase activity was measured by a microplate luminometer using the Bright-Glo luciferase assay system (Promega, WI, USA) following the manufacturer’s instruction. The percentage inhibition of MSTN activity was calculated by the following formula: Percentage of inhibition capability = (luminescence at ligands (1 nM MSTN, Activin A or GDF11)–luminescence at each truncated propeptide concentration) × 100 / (luminescence at ligands (1 nM MSTN, Activin A or GDF11)–luminescence at 0 nM ligands (MSTN, Activin A or GDF11). IC_50_ (ligand concentration inhibiting 50% of MSTN, GDF11 or Activin A activity) values were estimated by a non-linear regression model defining a dose-response curve as described previously [39] using a Prism6 program (GraphPad, CA, USA). The equation for the model was Y = Bottom + (Top-Bottom)/[1+10^(X-LogIC_50_)], where Y is % inhibition, Bottom is the lowest value of % inhibition set at 0%, Top is the highest value of % inhibition set at 100%, and X is Log ligand concentration. IC_50_ values were analyzed by ANOVA using the same program.

### Western blot analysis

The effect of MBP-Pro45-70-His6 on MSTN-induced phosphorylation of Smad2 and Smad3 was examined in HepG2 cells that induce Smad2 phosphorylation in the presence of MSTN [13]. HepG2 cells were seeded in 6-well plates at the density of 2×10^5^ cells per well and grown in DMEM containing 10% FBS, 1% penicillin/streptomycin at 37 °C with 5% CO_2_ for 24 h. Following serum-free DMEM containing 10% FBS, 1% penicillin/streptomycin for 4 h, cells were treated with or without 10 nM MSTN (R&D Systems, WI, USA), 10 μM SB431542, an inhibitor of activin receptor-like kinase (ALK)4/5/7 (Sigma, MA, USA) and 600 nM MBP-Pro45-70-His6 for 30 min. Then, cells were lysed in RIPA buffer (20 mM Tris-HCl, pH 7.5, 150 mM NaCl, 1 mM Na_2_EDTA, 1 mM EGTA, 1% NP-40, 1% sodium deoxycholate, 2.5 mM sodium pyrophosphate, 1 mM β-glycerophosphate, 1 mM Na_3_VO_4_, 1 μg/mL leupeptin (Cell Signaling, MA, USA) containing protease inhibitor cocktail and phosphatase inhibitor cocktail (Roche, NJ, USA). The lysates were disrupted by sonication for 15 s on the ice and centrifuged at 12,000 rpm for 20 min at 4 °C. Protein lysates were quantified by BCA assay (Thermo Scientific, MA, USA). Fifty μg of protein were separated by 10% polyacrylamide SDS-PAGE and transferred to polyvinylidene fluoride membranes (Millipore, MA, USA). The membranes were blocked with 5% nonfat or 5% BSA in Tris-buffered saline (TBS) containing 0.1% tween 20 (TTBS) for 3 h at room temperature. To assess phosphorylated and total protein of signaling molecules, the membranes were incubated with antibodies against respective phospho- or total protein for 3 h at room temperature. The following primary monoclonal antibodies and polyclonal antibody (β-actin) were used and purchased from Cell Signaling Technology (MA, USA): Smad2 (Cat#31221:1000), phospho-Smad2 (Cat#3108, 1:1000), Smad3 (Cat#9523,1:1000), phospho-Smad3 (Cat#9520,1:1000), β-actin (Cat#3700, 1:1000). The membranes were washed with TTBS for 10 min four times and then incubated for 3 h at room temperature with horseradish peroxidase-conjugated matching secondary antibodies (1:5000): anti-mouse IgG for Smad2, p-Smad2, Smad3, and p-Smad3; anti-Rabbit IgG for β-actin. The membranes were washed 3 times with TTBS for 10 min. The immuno-reactive bands were detected by the SuperSignal West Femto Maximum Sensitivity Substrate (Thermo Scientific, MA, USA) and visualized on Kodak Omat X-ray films. Densitometry analysis of the images obtained from X-ray films was performed using the Image J software (NIH).

### Animal experiments

ICR male mice were obtained from Hyochang Science (Seoul, Korea). We followed the current regulations for the care and use of laboratory animals under the guidance of the Animal Ethics Committee of Pukyong National University, Busan, Korea. The ethics committee approved this study under protocol AEC-201405. The mice were maintained in a room at a constant temperature of 24±1°C under a 12 h light/12 h dark cycle at 65% humidity. Animal pellet (Formula M07; FeedLab, Korea) and water were provided *ad libitum*. For the MBP-Pro45-70-His6 administration study, mice (5~6 week old weighing 24–26 g) were randomly divided into two groups (n = 12 per group). Animals were injected subcutaneously with 0 or 24 mg/kg body wt of MBP-Pro45-70-His6 five times (3 days apart) for 14 days. MBP-Pro45-70-His6 was dissolved in PBS (2 μg/μL) for mice in the treatment group, and PBS was injected for mice in the control group. Body wt was measured at the time of injection.

For the Pep45-65-NH2 administration study, mice (6~8 week old weighing 27–30 g) were randomly divided into two groups (n = 12 per group). Pep45-65-NH2 was dissolved in PBS (2 μg/μL), and subcutaneously injected (10 μL/g body wt) five times (3 days apart) for 14 days for mice in the treatment group. Mice in the control group were injected with PBS. Body wt was measured at the time of injection.

### Forelimb grip strength measurement

Forelimb grip strength was measured 1 h after each injection. The tensile force generated by each mouse was measured using a force transducer with a rectangular 4×5 cm^2^ metal net (Model-RX-10 Aikoh Engineering Co., Osaka, Japan). Briefly, the mouse was slowly pulled by the tail in the opposite direction while its two forelimbs gripped the metal net. The grip strength meter was recorded in Newton (N). Each mouse was subjected to three times grip test with at least a 1 min rest between trials. The maximum grip force was recorded as the forelimb grip strength. Grip strength (N) was measured on day 0 (before the first injection), day 7, and day 14.

### Swimming time measurement

The forced swimming test was performed 1 h after the grip strength measurement. For the forced swimming test, mice were dropped individually in a 1,000 mL beaker (25 cm in height, 10 cm in diameter) filled with water at 24±1 °C. After a 2 min adaptation period, the swimming time (s) was measured following the procedure described previously [40]. Another swimming time was measured at 4 h after the 1^st^ measurement.

### Analysis of blood glucose, cholesterol, triglyceride, and fatty acids

On day 14, the mice were anesthetized with Zoletil 50 (Virbac, Carros, France; 10 mg/kg, intramuscular), and blood was collected by cardiac puncture [41]. The blood, after clotting for 10 min on ice, was centrifuged at 3,000 rpm for 15 min to obtain serum and the serum was stored at -70°C until use. The serum levels of total cholesterol, triglyceride, and glucose were measured by SD LipidoCare system-analyzer (SD Biosensor, Seoul, Korea). The levels of free fatty acid (FFA, #K612-100) in serum were measured by colorimetric assay kits (Biovision, CA, USA).

### Skeletal muscle and bone mass measurement

After peeling the skin off the euthanized mice, forelimbs and hindlimbs were disarticulated from scapula to carpus and from ilium to medial malleolus, respectively. The wt of forelimbs and hindlimbs were immediately measured, as well as the bone wt. The skeletal muscle mass was calculated by subtracting the wt of the bone from those of the forelimbs and hindlimbs, respectively.

### ELISA for antibody titer against MBP-Pro45-70-His6

Blood for ELISA was collected on day 7 using the facial vein technique [42] and on day 14 by cardiac puncture. ELISA plates were coated with 100 μL of an MBP-pro45-70-His6 solution (1 μg/mL PBS) per well overnight at 4°C. The plates were washed three times with PBS, followed by blocking of the plate with 150 μL PBS containing 1% bovine serum albumin at room temperature for 3 h. The plates were washed three times with PBS and added with 100 μL diluted sera (1:1000 with blocking solution) in each well, then incubated at room temperature for 3 h. The plates were washed four times with PBS, then, 100 μL alkaline-phosphate conjugated anti-mouse IgG (Sigma, USA) diluted 1:5,000 with blocking solution was added to each well and incubated at room temperature for 3 h. After washing four times with PBS, 100 μL of p-Nitrophenyl Phosphate (Sigma, USA) liquid substrate solution was added to each well and incubated for 30 min at 37 °C in the dark. The absorbance was measured at 450 nm with a microplate reader (Biotek, Seoul, Korea). All samples were tested in triplicate.

## Results

### MSTN-inhibitory capacities of truncated forms of MBP-MSTNpro proteins

Fifteen truncated, MBP-fused MSTNpro proteins, including MBP-Pro45-70, MBP-Pro45-69, MBP-Pro45-68, MBP-Pro45-67, MBP-Pro45-66, MBP-Pro45-65, MBP-Pro45-64, MBP-Pro45-63, MBP-Pro45-62, MBP-Pro45-61, MBP-Pro45-60, MBP-Pro46-70, MBP-Pro46-69, MBP-Pro47-70, and MBP-Pro49-70 were expressed and purified by amylose affinity chromatography. Sequence analysis confirmed the amino acid sequences of each MSTN prodomain. The anti-MSTN activities were measured using the pGL3-(CAGA)_12_-luciferase reporter assay, and IC_50_ values for 1 nM MSTN inhibition were estimated (Table 1). MBP-Pro45-70 had the most potent MSTN-inhibitory capacity with an IC_50_ value of 1.18 nM, and removing 70 and 69 amino acids sequentially from the C-terminal side of Pro45-70 gradually diminished the MSTN-inhibitory capacity with IC_50_ values of 2.02 and 9.33 nM, respectively (Table 1). Removing 68, 67, and 66 sequentially from the C-terminal side of Pro45-70 drastically diminished the MSTN-inhibitory capacity with IC_50_ values of 0.2, 8.0 and 3.1 μM, respectively. Removing 65 and below from the C-terminal side of Pro45-70 abolished the MSTN-inhibitory capacity. Removing 45 and 46 sequentially from the N-terminal side of Pro45-70 diminished the MSTN-inhibitory capacity with IC_50_ values of 27.7and 10.9 nM respectively. Removal of 45 and 70 from the Pro45-70 also diminished the MSTN-inhibitory activity (IC_50_, 21.9 nM). Removal of 47 and 48 from the N-terminal side of Pro45-70 drastically diminished the MSTN-inhibitory capacity with IC_50_ values of 1.03 μM.

**Table 1:** The inhibition of MSTN activity by MBP-fused, truncated MSTN1 propeptides of flatfish and immunogenicity of the truncated MSTN1 propeptides.

Truncated MSTN1 propeptide	Sequence of propeptide	*IC_50_	Immunogenicity^+^
MBP-Pro45-70	CDVRQQIKTMRLNAIISQILSKLRMK	1.18 nM	+
MBP-Pro45-69	CDVRQQIKTMRLNAIISQILSKLRM	2.02 nM	+
MBP-Pro45-68	CDVRQQIKTMRLNAIISQILSKLR	9.33 nM	+
MBP-Pro45-67	CDVRQQIKTMRLNAIISQILSKL	0.2 μM	+
MBP-Pro45-66	CDVRQQIKTMRLNAIISQILSK	8.0 μM	+
MBP-Pro45-65	CDVRQQIKTMRLNAIISQILS	3.1 μM	-
MBP-Pro45-64	CDVRQQIKTMRLNAIISQIL	ND	-
MBP-Pro45-63	CDVRQQIKTMRLNAIISQI	ND	-
MBP-Pro45-62	CDVRQQIKTMRLNAIISQ	ND	-
MBP-Pro45-61	CDVRQQIKTMRLNAIIS	ND	-
MBP-Pro45-60	CDVRQQIKTMRLNAII	ND	-
MBP-Pro46-70	DVRQQIKTMRLNAIISQILSKLRMK	27.65 nM	+
MBP-Pro46-69	DVRQQIKTMRLNAIISQILSKLRM	21.89 nM	+
MBP-Pro47-70	VRQQIKTMRLNAIISQILSKLRMK	10.94 nM	+
MBP-Pro49-70	QQIKTMRLNAIISQILSKLRMK	1.03 μM	+
MBP-Pro45-70-His6**	CDVRQQIKTMRLNAIISQILSKLRMK-His6	1.43 nM	+
Pep45-65-NH2***	CDVRQQIKTMRLNAIISQILS-NH2	2.66 μM	-

HEK293 cells stably expressing (CAGA)_12_-luciferase gene construct were used to measure MSTN-inhibitory capacity.

^+^Immunogenicity of the MSTNpro fragment without MBP fusion partner was determined using the method of Kolaskar and Tongaonkar (1990) (http://imed.med.ucm.es/Tools/antigenic.pl).

*IC_50_ for 1 nM MSTN inhibition;

**Hisx6 tag was fused to the C-terminal side of the recombinant protein for further purification; ND, not detectable;

***The peptide was synthesized by Peptron Co. (Korea).

**Fig 1:**
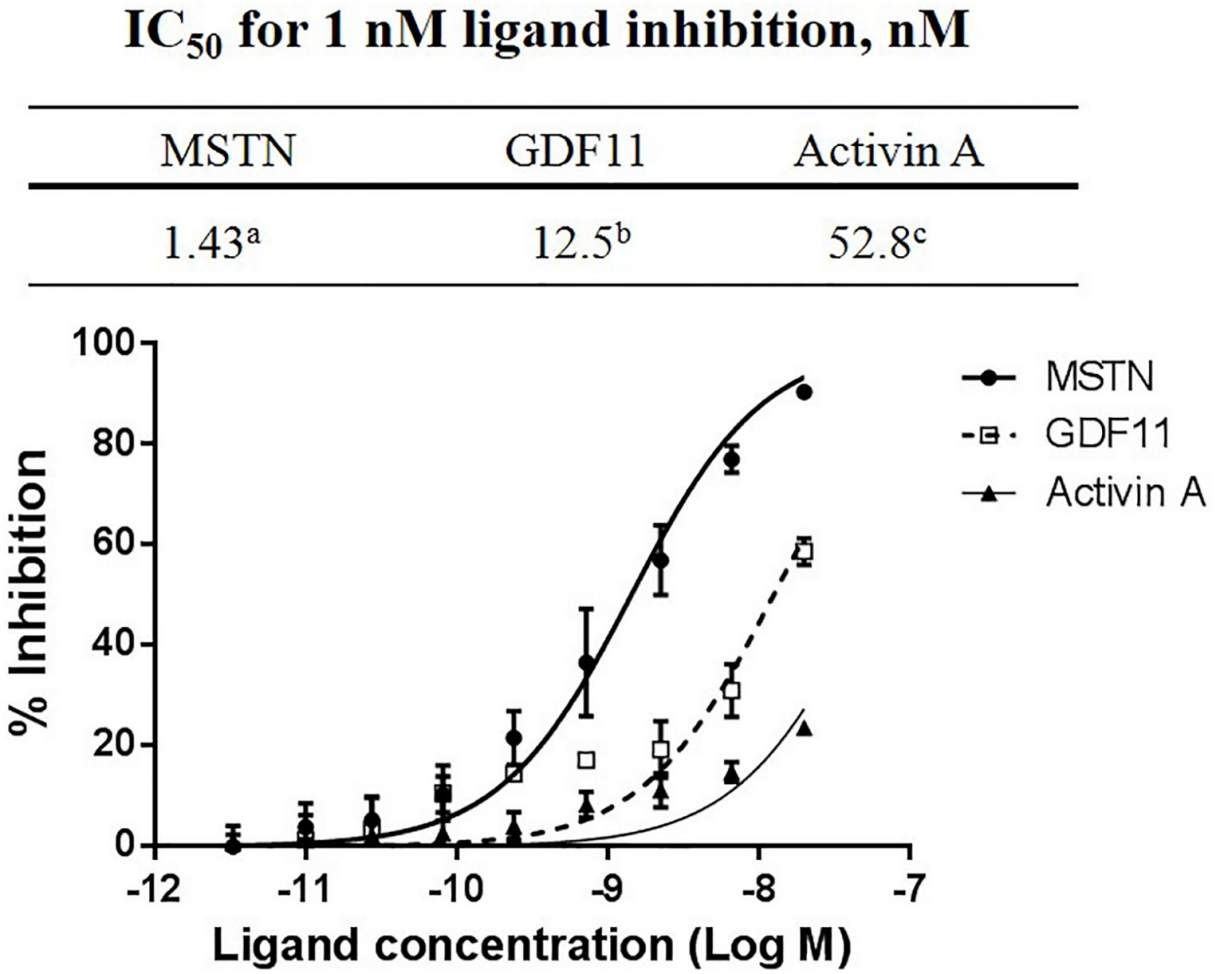
MBP-Pro45-70-His6 inhibits MSTN activity at much lower concentration than those required to inhibit GDF11 or Activin A activities. Various concentrations (20, 6.67, 2.22, and 0.73 nM) of MBP-Pro45-70-His6 in combination with 1 nM MSTN, GDF11, or Activin A in serum-free DMEM were added to the HEK293 cells stably expressing (CAGA)_12_-luciferase gene construct, followed by incubation for 24 h. Luciferase activity was measured using Bright-Glo luciferase assay system (Promega, USA). The error bars indicate standard deviation (n = 3). Percentage of inhibition capacity = (luminescence at ligands (1 nM MSTN, GDF11, or Activin A)–luminescence at each MBP-Pro45-70-His6 concentration) × 100/(luminescence at ligands (1 nM MSTN, GDF11, or Activin A)–luminescence at 0 nM ligands. IC_50_ (ligand concentration inhibiting 50% of MSTN, GDF11 or Activin A activity) values were estimated by a non-linear regression model defining the dose-response curve. IC_50_ values not sharing the same superscript are different at P<0.01.

The His6 tag was inserted at the C-terminus of Pro45-70 to facilitate further purification and to examine the effect of the presence of His6 tag on MSTN-inhibitory capacity and its administration on muscle growth and performance in mice. SDS-PAGE analysis of the MBP-Pro45-70-His6 during the two-step purification revealed the production of MBP-Pro45-70-His6 with very high purity (S1 Fig). The IC_50_ value of MBP-Pro45-70-His6 was 1.43 nM for 1 nM MSTN inhibition (Fig 1), which is very close to that of MBP-Pro45-70 (1.18 nM). To examine the extent of specificity of MBP-Pro45-70-His6’s binding to MSTN, the inhibitory activities of MBP-Pro45-70-His6 against GDF11, structurally very similar member to MSTN, and Activin A, another TGF-beta family member, were measured. IC_50_ values of MBP-pro45-70-His6 for 1 nM GDF11 (12.5 nM) and Activin A (52.8 nM) were about 8-fold and 37-fold higher, respectively than for 1 nM MSTN (Fig 1), indicating that MBP-Pro45-70-His6 is highly specific in suppressing MSTN activity.

### MBP-Pro45-70-His6 blocks Smad2/3 phosphorylation induced by MSTN

**Fig 2:**
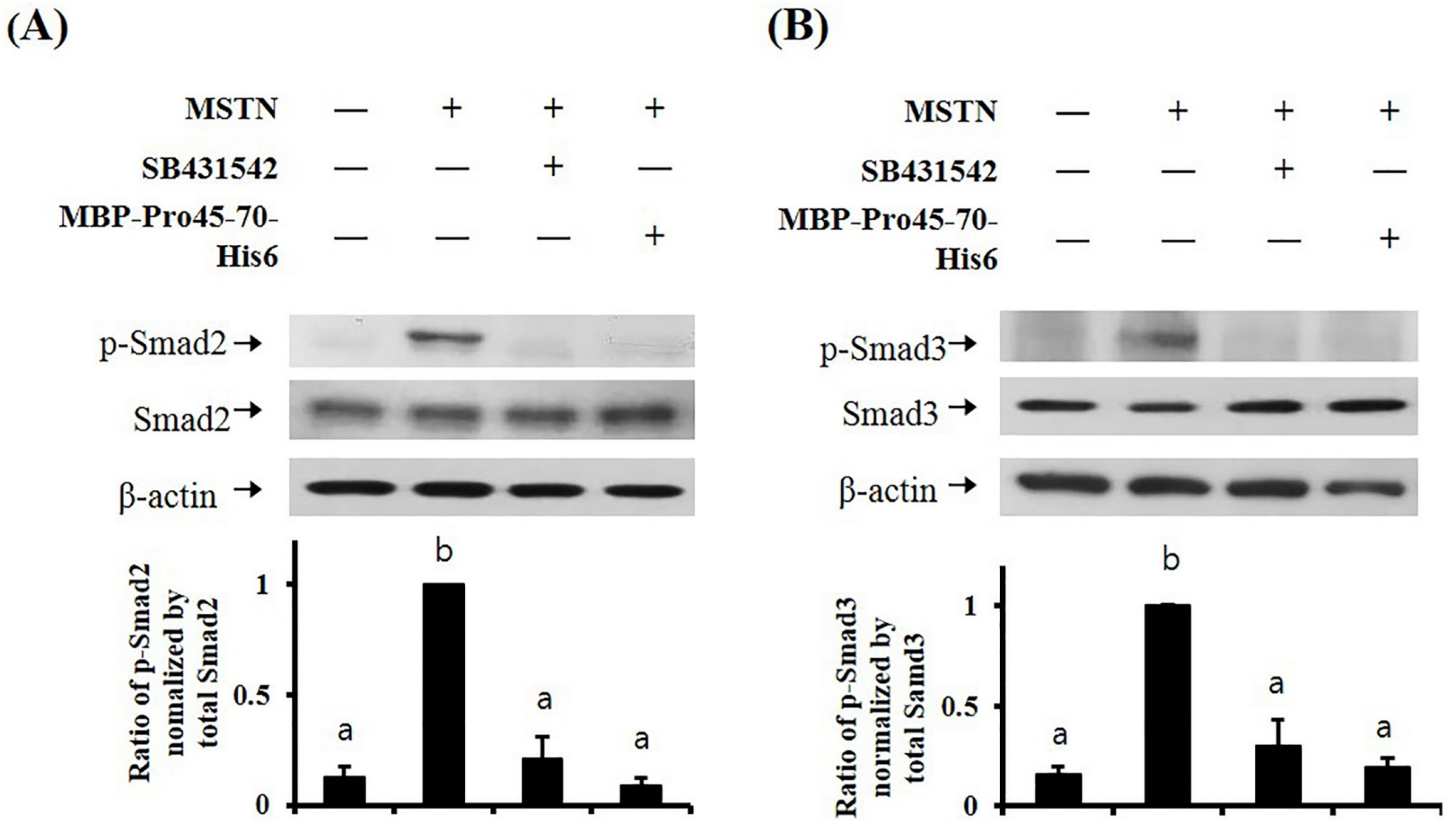
MBP-Pro45-70-His6 blocks MSTN-induced Smad2/3 phosphorylation in HepG2 cell. HepG2 cells were cultured in serum-free DMEM for 4 h, followed by treatment with the combination of 10 nM MSTN, 10 μM SB431542, or 600 nM MBP-Pro45-70-His6 for 30 min. The blots are representative of three independent assays and have been sequentially probed with antibodies against phospho-Smad2 (p-Smad2) or phospho-Smad3 (p-Smad3), total Smad2 (Smad2) or total Smad3 (Smad3), and b-actin. Densitometry analyses of the blots were from three independent assays. The Relative phosphorylation levels of p-Smad2 and p-Smad3 were normalized by the expression level of total Smad2 (A) or total Smad3 (B), respectively. The error bars indicate standard deviation (n = 3). Different letters are significantly different at *P*<0.05.

MSTN binds to specific serine/threonine kinase transmembrane receptor type IIB (ActRIIB) and induces phosphorylation of activin receptor-like kinase4 (ALK4) or activin receptor-like kinase5 (ALK5) type I receptor [43]. The MSTN/receptors complex allows intracellular signal propagation via phosphorylation of the Smad transcription factor proteins. Thus, we examined the phosphorylation level of Smad2 and Smad3 in HepG2 cells treated with MSTN plus MBP-Pro45-70-His6. We used MBP-Pro45-70 in this experiment because it showed the highest MSTN-inhibitory potency, indicating that less amount will be required in animal studies for MSTN inhibition among the truncated MSTN propeptides. In the control group without any treatment, the phosphorylation of Smad2 and Smad3 were not detected, while MSTN treatment induced phosphorylation of Smad2 and Smad3. Both MBP-Pro45-70-His6 and SB431542, an ALK5 inhibitor, blocked the MSTN-induced phosphorylation of Smad2 and Smad3, indicating that MBP-Pro45-70-His6 suppresses the Smad signaling pathway induced by MSTN (Fig 2).

### MBP-Pro45-70-His6 administration does not affect body wt growth but increases muscle wt, forelimb grip strength, and swimming time

**Fig 3:**
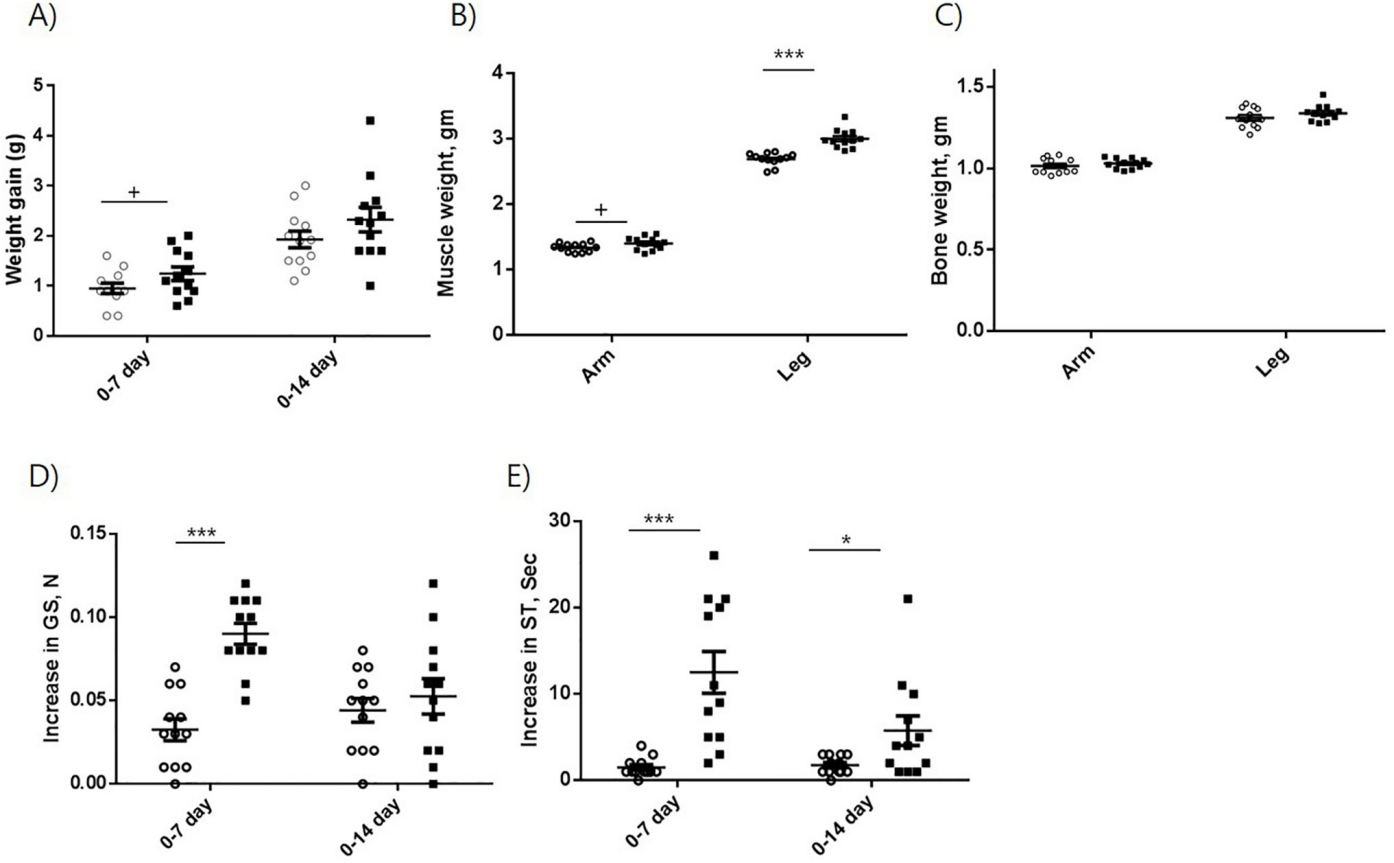
Effects of MBP-Pro45-70-His6 on body wt gain (A), grip strength (B), swimming time (C), muscle wt (D), and bone mass (E). Open circle (○) and closed square (■) indicate 0 and 20 mg/kg body wt administration of MBP-Pro45-70-His6, respectively. Student`s *t*-test was used to compare the mean difference. +, P<0.1; *, P<0.05; **, P<0.01; ***. P<0.001.

The administration of MBP-Pro45-70-His6 tended to increase the body wt gain during 7 days administration period as compared to the control, but no difference in body wt gain was observed after 14 days administration (Fig 3A). The muscle wts of forelimb (1.40 g) and hindlimb (3.00 g) of treated mice were significantly heavier than those (1.34 g and 2.69 g, respectively) of control mice after 14 days administration (Fig 3B). The bone wts of both forelimb and hindlimb were not affected by the MBP-pro45-70-His6 administration (Fig 3C). Apparently, the increase in muscle wt did not affect the body wt gain. Since suppression of MSTN is known to decrease the accumulation of adipose tissue [44], it is speculated that fat mass of the treated group was less than that of the control group, resulting in no difference in body wt gain. The effect of MBP-Pro45-70-His6 on forelimb grip strength was examined by measuring the change in grip strength from day 0 to day 7 and from day 0 to day 14 (Fig 3D). The mice treated with MBP-pro45-70-His6 had a significantly greater increase in grip strength from day 0 to day 7 than the control mice (0.090 N vs 0.0325 N). However, the increase in grip strength of the mice administered with MBP-pro45-70-His6 from day 0 to day 14 (0.0442 N) was not different from that of control mice (0.0525 N).

The effect of MBP-pro45-70-His6 on swimming time was also examined by measuring the change in swimming time from day 0 to day 7 and from day 0 to day 14 (Fig 3E). The mice treated with MBP-pro45-70-His6 had 8-fold greater increase in swimming time from day 0 to day 7 than the control mice (12.5 s vs 1.5 s), while the increase in swimming time by MBP-pro45-70-His6 from day 0 to day 14 was about 3-fold greater than that of control mice (5.75 s vs 1.75 s).

### MBP-Pro45-70-His6 administration suppresses blood cholesterol, triglyceride, and free fatty acids concentrations

The concentrations of serum cholesterol, triglyceride, and free fatty acid of mice administered with MBP-Pro45-65-His6 were significantly lower than those of control mice, while no difference in serum glucose concentration was observed between the two groups (Table 2).

**Table 2:** Effects of MBP-Pro45-70-His6 on blood glucose, total cholesterol, triglyceride, and free fatty acid level on day 14.

Group	Glucose (mg/dl)	Total cholesterol (mg/dl)	Triglyceride (mg/dl)	Free fatty acid (mM)
Control	371.3±18.56	150.8±4.27	180.0±6.55	1.101±0.056
MBP-Pro45-70-His6	335.3±15.08	126.2±5.86*	106.2±9.54***	0.758±0.063**
Relative level (%)	90	84	59	69

Relative levels are expressed as percentages of the values to the saline control group. Values are means ± SEM (n = 6). Student`s *t*-test was used to compare the mean difference.

*p<0.05;

**p<0.01;

***p<0.001.

### MBP-Pro45-70-His6 administration induced MBP-Pro45-70-His6 antibody production

**Fig 4:**
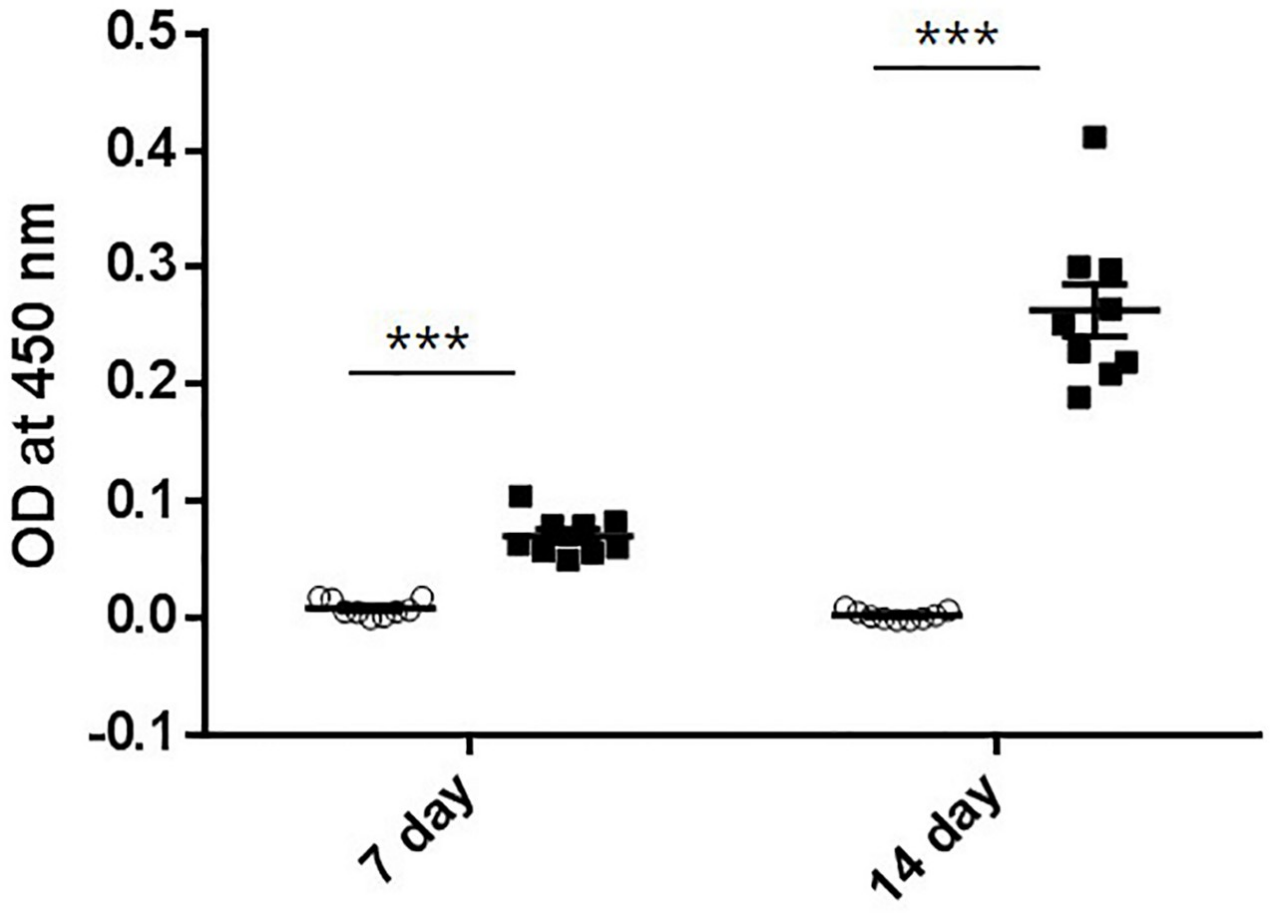
Titer values after the administration of MBP-Pro45-70-His6. 1000-fold diluted sera were used for titer measurement. Open circle (○) and closed square (■) indicate 0 and 20 mg/kg body wt administration of MBP-Pro45-70-His6, respectively. Student`s *t*-test was used to compare the mean difference. ***. P<0.001.

MBP-Pro45-70-His6 is a heterologous protein to mice, thus the generation of antibody against MBP-Pro45-70-His6 was examined using ELISA (Fig 4). The presence of an antibody against MBP-Pro45-70-His6 was observed at both 7 and 14 days after the administration with the titer value at day14 being significantly greater than that at day 7.

### Pep45-65-NH2 suppresses MSTN activity, but not the activities of either GDF11 or Activin A

**Fig 5:**
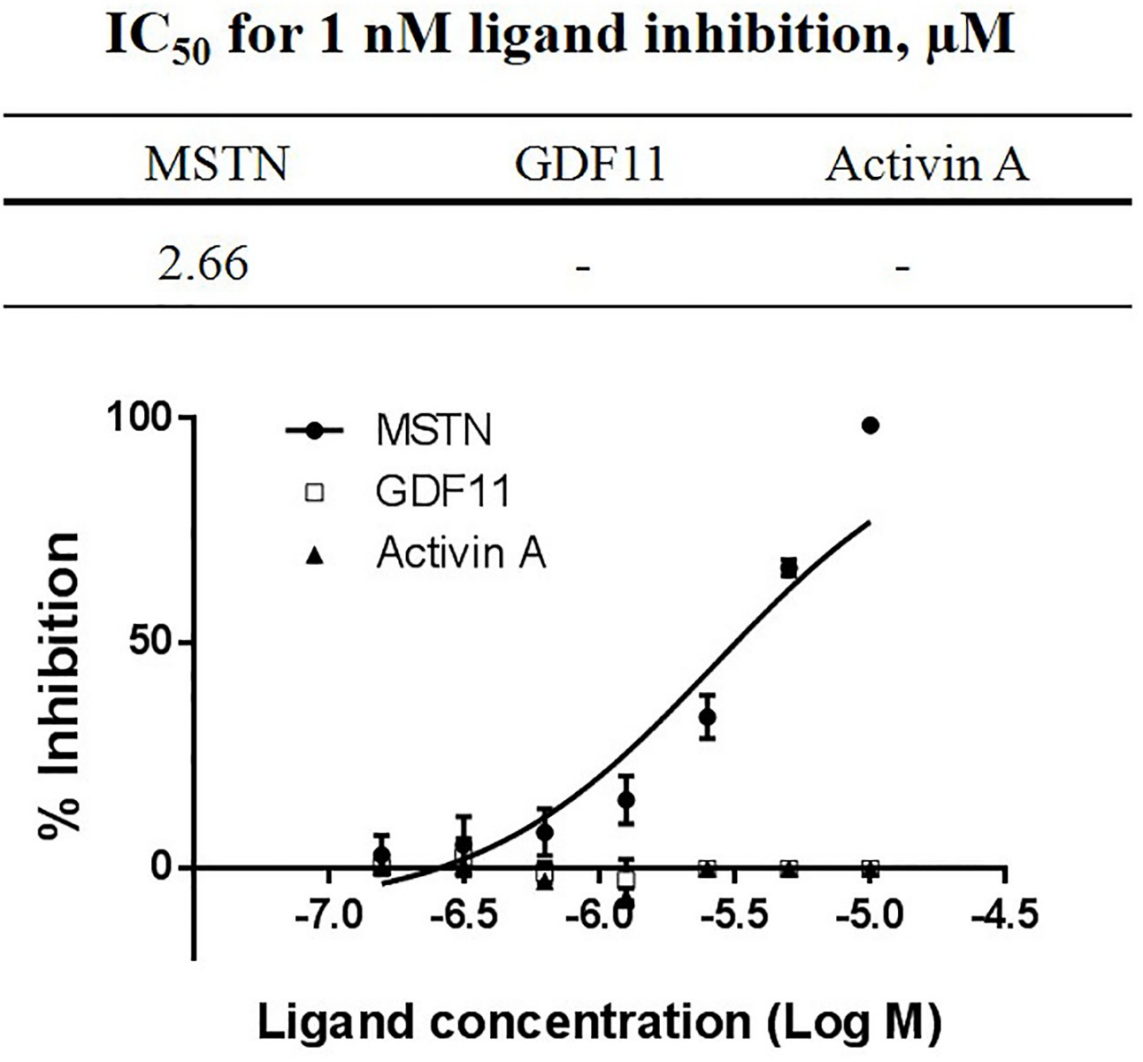
Inhibition of MSTN, Activin A, and GDF11 activities by Pep45-65-NH2. Various concentrations of Pep45-65-NH2 (10, 5, 2.5, 1.25, 0.625, 0.313, and 0.156 μM) in combination with 1 nM MSTN, GDF11 or Activin A were added to the HEK293 cells stably expressing (CAGA)_12_-luciferase gene construct, followed by incubation for 24 h. Luciferase activity was measured using Bright-Glo luciferase assay system (Promega, USA). The error bars indicate standard deviation (n = 3). Percentage of inhibition capacity = (luminescence at ligands (1 nM MSTN, GDF11, or Activin A)–luminescence at each MBP-Pro45-70-His6 concentration) × 100/(luminescence at ligands (1 nM MSTN, GDF11, or Activin A)–luminescence at 0 nM ligands. IC_50_ (ligand concentration inhibiting 50% of MSTN) values were estimated by a non-linear regression model defining the dose-response curve.

When the capacity of Pep45-65-NH2 to suppress 1 nM MSTN was examined with the concentration range of Pep45-65-NH2 between 100 nM and 10 μM, Pep45-65-NH2 demonstrated MSTN-inhibitory capacity with the IC_50_ value of 2.66 μM (Fig 5). However, in this rage of ligand concentration, Pep45-65-NH2 did not suppress 1 nM GDF11 or Activin A (Fig 5), indicating that Pep45-65-NH2 is highly specific in suppressing MSTN activity.

### Pep45-65-NH2 blocks Smad2 phosphorylation induced by MSTN

**Fig 6:**
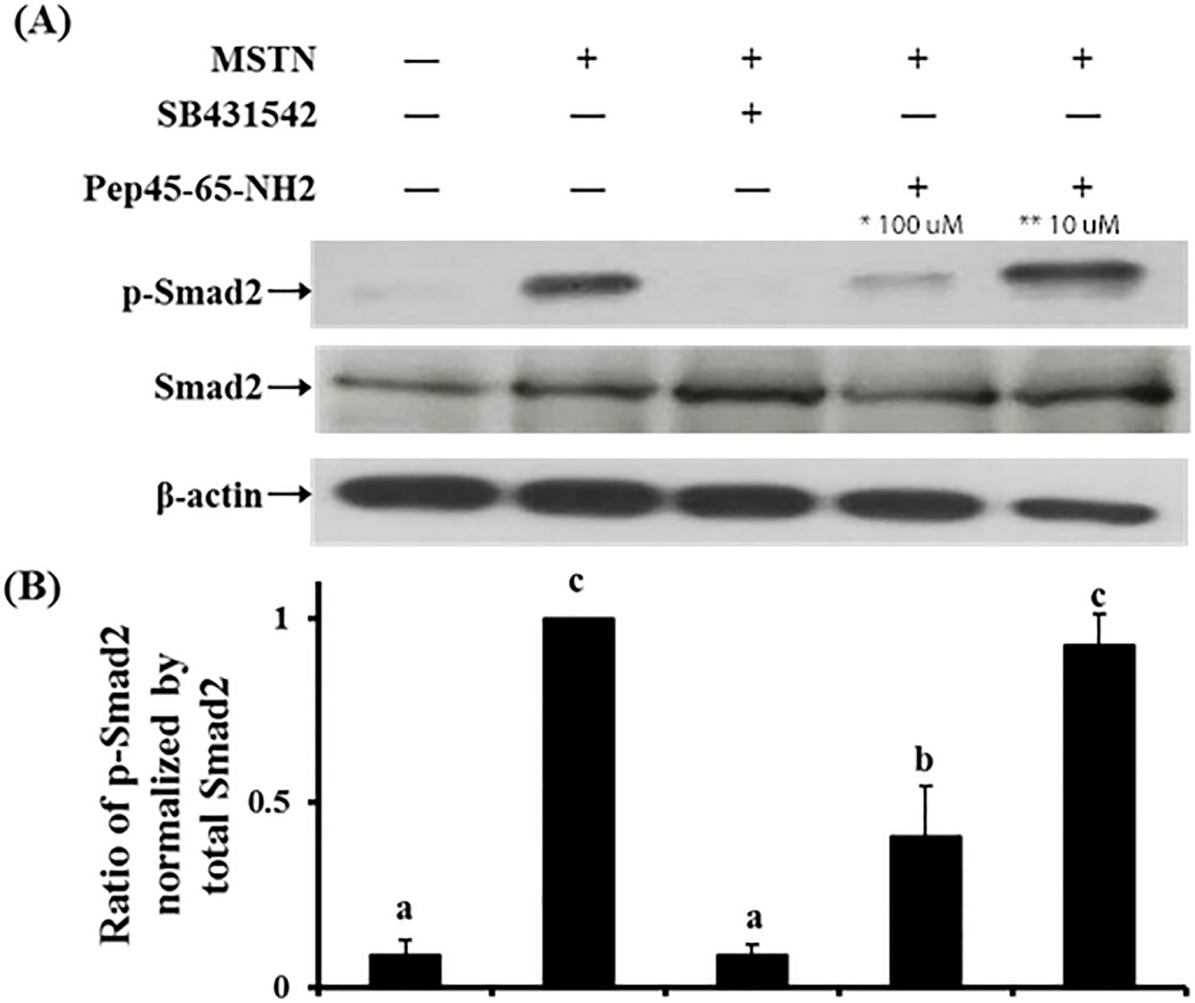
Effects of Pep45-65-NH2 on myostatin-induced Smad2 phosphorylation in HepG2 cells. (A) HepG2 cells were cultured in serum-free DMEM for 4 h, followed by treatment absence and/or presence 10 nM MSTN, 10 μM SB431542, and 100 μM and 10 μM Pep45-65-NH2 for 30 min. The blots are representative of three independent assays and have been sequentially probed with antibodies against phospho-Smad2 (p-Smad2), total Smad2 (Smad2), and b-actin. (B) Densitometry analyses of the blots were from three independent assays. The Relative phosphorylation level of p-Smad2 was normalized by the expression level of total Smad2. The error bars indicated standard deviation (n = 3). Different letters are significantly different at *P*<0.01. *,100 μM concentration of Pep45-65-NH2; **, 10 μM concentration of Pep45-65-NH2.

The MSTN-induced Smad2 phosphorylation was not blocked by the 10 μM Pep45-65-NH2 but almost completely blocked by 100 μM Pep45-65-NH2, demonstrating a dose-dependent suppression of MSTN activity by Pep45-65-NH2 (Fig 6).

### Pep45-65-NH2 administration enhances body wt growth, muscle wt, forelimb grip strength, and swimming time

**Fig 7:**
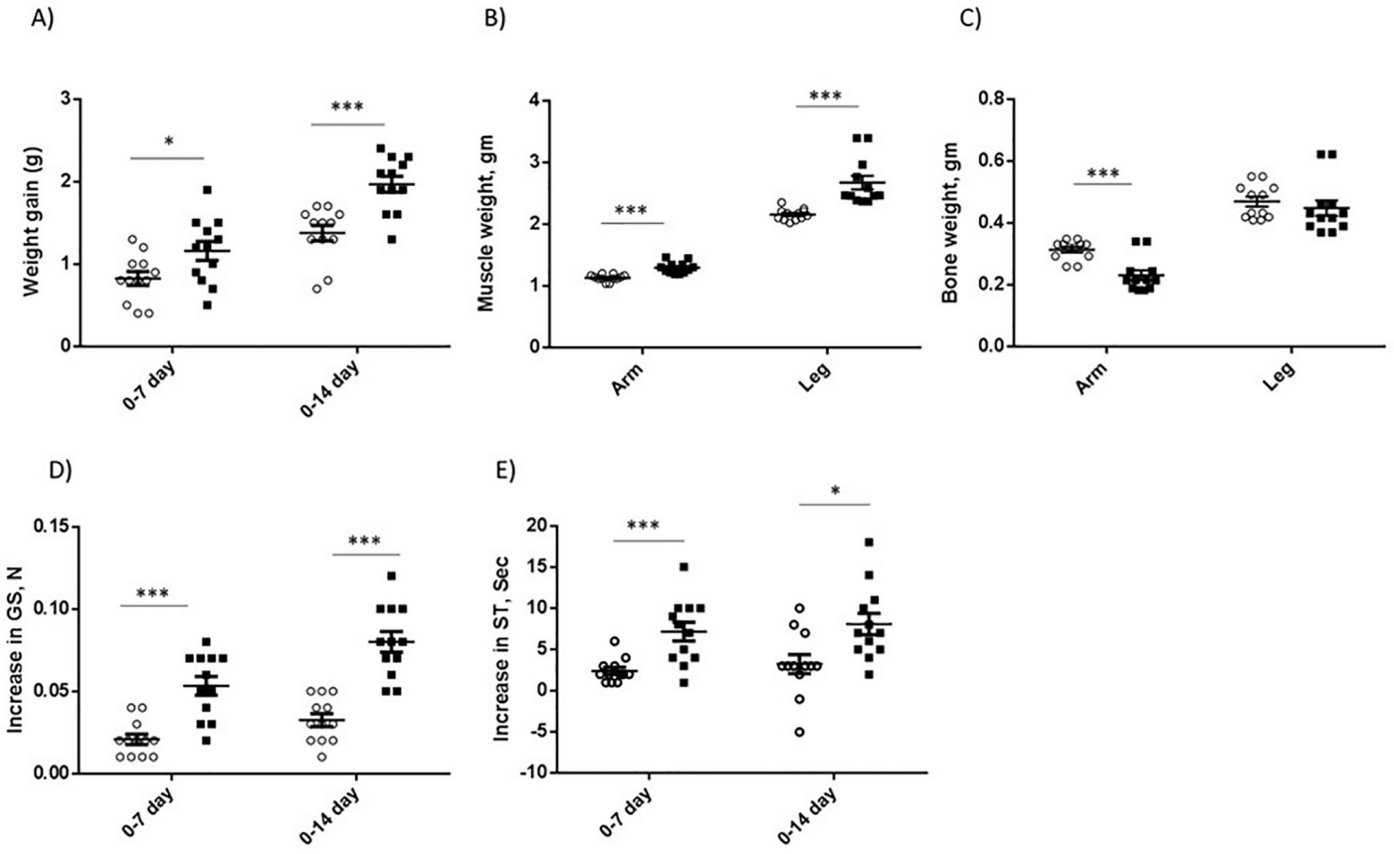
Effects of Pep45-65-NH2 on body wt gain (A), muscle wt (B), bone mass (C), increase in grip strength (D), and increase in swimming time (E). Open circle (○) and closed square (■) indicate 0 and 20 mg/kg body wt administration of Pep-45-65-NH2. Student`s *t*-test was used to compare the mean difference. +, P<0.1; *, P<0.05; **, P<0.01; ***. P<0.001.

The administration of Pep45-65-NH2 increased the body wt gain during the 7 and 14 days administration period as compared to the control (Fig 7A). The increase in gain at day 14 (0.809 g) was greater than that at day 7 after administration (0.55 g), suggesting that the increase in body wt gain by Pep45-65-NH2 continued during the 14 days administration. The muscle wts of forelimb (1.30 g) and hindlimb (2.67 g) of treated mice were significantly heavier than those (1.14 g and 2.16 g, respectively) of control mice after 14 days administration (Fig 7B). Forelimb bone wt of the treatment group was significantly lower than that of the control group after 14 days administration, but no difference in hindlimb bone wt was observed between the two groups (Fig 7C).

The mice treated with Pep45-65-NH2 had a greater increase in grip strength from day 0 to day 7 than the control mice (0.0533 N vs 0.0208 N), as well as from day 0 to day 14 (0.0800 N vs 0.0325 N) (Fig 7D). The mice treated with Pep45-65-NH2 had a greater increase in swimming time from day 0 to day 7 than the control mice (7.17 s vs 2.42 s), as well as from day 0 to day 14 (8.08 s vs 3.25 s) (Fig 7E).

### Pep-45-65-NH2 administration suppresses blood glucose, triglyceride, and free fatty acids concentrations

The concentrations of serum glucose, triglyceride, and free fatty acid of mice administered with Pep45-65-NH2 were significantly lower than those of control mice, while no difference in serum cholesterol concentration was observed between the two groups (Table 3).

**Table 3:** Effects of Pep45-65-NH2 on blood glucose, total cholesterol, triglyceride, and free fatty acid level on day 14.

Group	Glucose (mg/dl)	Total cholesterol (mg/dl)	Triglyceride (mg/dl)	Free fatty acid (mM)
Control	354.5±8.20	140.7±4.52	131.7±8.46	0.572±0.108
Pep45-65-NH2	286.8±5.34*	136.7±5.96	96.8±4.78**	0.323±0.091
Relative activity (%)	80.9	97.1	73.5	56.4

Relative activities are expressed as percentages of the values in the negative control group. Values are means ± SE (n = 6).

*p<0.001 and

**p<0.005 compared with the negative control. Statistical analyses were performed using Student`s *t* test.

## Discussion

Myostatin propeptide (MSTNpro) is a well-known MSTN suppressor, and the administration of recombinant MSTNpro enhanced skeletal growth or muscle repair in mice [9, 11, 27, 32], demonstrating its potential as a pharmaceutical agent to treat various muscle wasting conditions or diseases. Recent studies have shown that synthetic peptides containing 29 or 24 amino acids from the mouse MSTNpro were sufficient to suppress MSTN activity in vitro and to increase muscle mass in mice of muscular dystrophy model [12, 13]. We also have shown that truncated forms of pig and flatfish MSTNpro with an N-terminal fusion of maltose binding protein (MBP) had the MSTN-inhibitory capacity not different from the full sequence mouse MSTNpro [35, 36]. Notably, the size of MSTNpro region of flatfish (residues 45–80) was much smaller than that of pig (residues 42–175) for full MSTN-inhibitory capacity, suggesting that the large sequence variation between fish and mammalian MSTNpro affects the stability of MSTNpro and MSTN complex.

**Fig 8:**
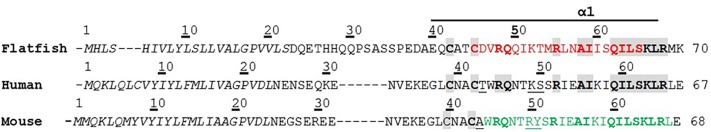
Amino acid sequences from 1 to 70 of flatfish myostatin propeptide aligned with those of mouse and human. The italic characters indicate signal sequence. The bold shaded amino acid residues indicate the same sequence identity within the α1 helix region among the three species. The underlined amino acid sequences indicate different sequences within the α1 helix region between mouse and human. The α1 helix domain is based on Shi et al. [46].

The current study shows that MSTN-inhibitory capacity (IC_50_, 1.18 nM) of flatfish MSTNpro region consisting of residues 45–70 with MBP fusion (MBP-MSTNpro45-70) is not different from that of MBP-MSTNpro-45-80 (IC50, 1.30 nM) reported previously [36]. Removing the residue 70 or 70 plus 69 did not drastically affect the MSTN inhibitory capacity, indicating that flatfish MSTNpro region 45–68 with MBP as a fusion partner is sufficient to have full MSTN-inhibitory capacity and residues beyond 68 contribute little to the stability of MSTNpro and MSTN complex. The flatfish MSTNpro region 45–70 is almost homologous to mouse MSTN region 44–67 (Fig 8), which was reported as a minimum domain for MSTN suppression [13]. The region is in the α-helix forming domain of MSTNpro (Fig 8). A recent study on a crystal structure of human unprocessed MSTN showed that the region from Arg45 to Leu 64 forms α-helical structure, and six aliphatic residues in the helical structure are buried within the hydrophobic groove of an MSTN domain that is known to interact with MSTN type I and II receptors [45], supporting the essential role of this domain on MSTN inhibition.

Since the in vivo effect of MBP-fused MSTNpro has not been determined, we examined the effects of the administration of His6-fused MBP-MSTNpro45-70 for 2 weeks on muscle growth and performance. Muscle mass, grip strength, and swimming time were significantly enhanced by the administration, suggesting the inhibition of MSTN activity in vivo by MBP-MSTNpro45-70-His6. In support of the postulation of MSTN inhibition by MBP-MSTNpro45-70-His6 in vivo, MBP-MSTNpro45-70-His6 suppressed the MSTN-induced Smad2/3 phosphorylation in HepG2 cells. Notably, the increase in grip strength observed at week 1 disappeared at week 2 after the administration. Similarly, the increase in swimming time at week 2 was significantly less than at week 1 after the administration, suggesting that the effect of MBP-MSTNpro45-70-His6 diminishes or disappears during prolonged administration. Since MBP is a heterologous protein, it was suspected that immunogenic responses would occur against MBP-MSTNpro45-70-His6, leading to neutralization of the MSTN-suppressive effect of MBP-MSTNpro45-70-His6. The much greater titer value against MBP-Pro45-70-His6 from week 2 sera than that of week 1 sera indicates the neutralization of MBP-MSTNpro45-70-His6 by antibody formation against the recombinant protein.

A peptide fragment bearing flatfish MSTNpro residues from 45–65 (Pep45-65), which suppressed MSTN activity, was predicted to induce little immunogenicity in silico assay. Pep45-65 suppressed the MSTN-induced Smad2/3 phosphorylation dose-dependently in HepG2 cells, suggesting its potential to suppress MSTN activity *in vivo*. In mice experiment, the administration of Pep45-65 significantly enhanced body wt gain, muscle mass, grip strength, and swimming time. Unlike the administration of MBP-MSTNpro45-70-His6, the extent of increase in body wt gain and grip strength at week 2 was greater than at week 1, indicating that the effect of Pep45-65 on muscle growth and performance was accumulating during the prolonged administration. No antibody titer was detected against Pep45-65 (S2 Fig), suggesting that the non-mammalian nature of the peptide would probably cause little side effects that may arise from the antigenic response to heterologus peptide administration in mammalian species. Furthermore, Pep45-65 did not suppress GDF11, and Activin A in the concentration range tested. Given that MSTN and GDF11 share 89% homology and signal through the same transcription factors [1], the administration of Pep45-65 is likely to invoke minimal non-targeted effects *in vivo*. These results together, thus, point to the importance of the immunogenicity of recombinant proteins or peptides as pharmaceutical agents and indicate that Pep45-65 has a great therapeutic potential to treat muscle atrophy caused by various conditions and diseases.

In addition to its effect on muscle mass, MSTN also regulates fat mass and energy metabolism. Studies have shown that the inhibition of MSTN decreases fat mass, improves insulin sensitivity and whole-body metabolism in mice [47–52], and pig [53]. MSTN-suppression increased the expression of PGC-1α, a key regulator of mitochondrial biogenesis, brown adipocyte differentiation, and fatty acid metabolism, in the muscle and adipose tissue [49, 50, 53], indicating that the changes in metabolic profiles affected by MSTN-suppression are associated with the upregulation of PGC-1α. Recent studies also have shown that the serum level of Irisin, a myokine inducing white adipocyte browning and enhancing energy expenditure [54], was increased by MSTN suppression [50, 53, 55], suggesting a presence of cross talk between muscle and adipocyte in improving insulin sensitivity and enhancing energy metabolism by MSTN suppression. According to Dong et al. [50], MSTN-suppression increases PGC-1α expression and Irisin production in skeletal muscle, leading to the stimulation of white adipocyte browning and consequently enhanced energy expenditure and improved insulin sensitivity. In this study, the administration of MSTN suppressors (MBP-MSTNpro45-70-His6 and Pep45-65) lowered blood concentrations of glucose, triglyceride, and free fatty acids. In agreement with this result, others also reported that MSTN suppression lowers blood glucose, triglyceride, or fatty acids levels [47, 49, 53]. It is, thus, likely that energy metabolism was enhanced by the administration of MBP-MSTNpro45-70-His6 or Pep45-65, and future studies need to examine the potentials of these compounds to improve insulin sensitivity.

The size of Pep45-65 is smaller than that of mouse MSTNpro peptide fragment consisting of residues from 44 to 66 (p23) that was reported to be the minimum peptide for MSTN inhibition [13]. In spite of the smaller size the MSTN-inhibitory capacity of flatfish Pep45-65 appears to be comparable to or better than that of p23. The IC_50_ value of Pep45-65 for MSTN inhibition (12.5 ng/mL) was about 3 μM in our current and a previous study [36], while the IC_50_ value of p23 for MSTN inhibition (10 ng/mL) was reported to be 4.1 μM in a (CAGA)_12_ reporter gene assay [13]. The removal of residues from 64 to 66 (Leu-Arg-Leu) from p23 failed to show MSTN-inhibitory capacity [13], suggesting an important role of these sequences for effective inhibition of MSTN activity. However, our result shows that the region in flatfish MSTNpro may not be necessary for an effective MSTN suppression because in the absence of the corresponding region (67Leu-Arg-69Met), Pep45-65 was effective in suppressing MSTN activity. There are many other subtle differences in amino acid sequence between Pep45-65 and p23 (Fig 8), and future studies need to investigate the contributions of the differences to the interaction of MSTNpro peptides and MSTN and subsequence MSTN inhibition.

In conclusion, current results indicate that MSTN-inhibitory proteins with heterologous fusion partner may not be effective in suppressing MSTN activity in vivo due to an immune response against the proteins. Current results also show that the region of flatfish MSTNpro consisting of 45–65 (Pep45-65-NH2) can suppress MSTN activity and increase muscle mass and function without invoking an immune response in the mouse. The Pep45-65-NH2 with its smaller size than the mouse propeptide (p23, aa 44–66) showed a comparable to or better MSTN-inhibitory potency than the mouse propeptide. Also, Pep45-65-NH2 did not interfere with the GDF11 and Activin A signaling, suggesting minimal off-target effect *in vivo*. These results, together, imply that Pep45-65-NH2 would be a potential agent to enhance skeletal muscle growth and function in animals or to treat muscle atrophy caused by various clinical conditions.

## Supporting information

S1 Table Primers used for pMALc5x expression vector construction of truncated MSTN1 propeptides. Underline indicates EcoRI; site used in the construction of expression vectors.
(PDF)

S1 Fig SDS-PAGE analysis of MBP-Pro45-70-His6 purified with two-step chromatography. Ni-NTA agarose affinity chromatography was first used, followed by amylose resin affinity chromatography for purification. Proteins were visualized with Coomassie blue staining. **M,** protein ladder; **T**, total protein; **S**, soluble fraction; **P**: pellet (insoluble fraction), **FT1**: flow-through fraction of Ni-NTA agarose, **W1**: washing fraction of Ni-NTA agarose, **E1**: elution fraction of Ni-NTA agarose, **FT2**: flow-through fraction of amylose resin, **W2**: washing fraction of amylose resin, **E2**: elution fraction of amylose resin.
(TIF)

S2 Fig Titer values after the administration of Pro45-65-NH2. 1000-fold diluted sera were used for titer measurement. Open circle (○) and closed square (■) indicate 0 and 20 mg/kg body wt administration of Pro45-65-NH2, respectively. Student`s t-test was used to compare the mean difference.
(TIF)
