# Supplementary material for: Identification of the minimum region of flatfish myostatin propeptide (Pep45-65) for myostatin inhibition and its potential to enhance muscle growth and performance in animals
Source: PLoS One. 2019 Apr 18;14(4):e0215298. doi: 10.1371/journal.pone.0215298 (PMC6472743; doi:10.1371/journal.pone.0215298)
Supplement: S1 Table — Underline indicates EcoRI; site used in the construction of expression vectors. (PDF) [file pone.0215298.s001.pdf]

Truncated MSTN1 propeptide	Forward primer	Reverse primer
pMAL-c5x-Pro45-70	TGA GAA TTC CCT GCA GGT	CTT CAT TCG CAG TTT GCT C
pMAL-c5x-Pro45-69	TGA GAA TTC CCT GCA GGT	CAT TCG CAG TTT GCT CAG
pMAL-c5x-Pro45-68	TGA GAA TTC CCT GCA GGT	TCG CAG TTT GCT CAG AAT C
pMAL-c5x-Pro45-67	TGA GAA TTC CCT GCA GGT	CAG TTT GCT CAG AAT CTG AG
pMAL-c5x-Pro45-66	TGA GAA TTC CCT GCA GGT	TTT GCT CAG AAT CTG AGA TTT
pMAL-c5x-Pro45-65	TGA GAA TTC CCT GCA GGT	GCT CAG AAT CTG AGA TTT GAT C
pMAL-c5x-Pro45-64	TGA GAA TTC CCT GCA GGT	CAG AAT CTG AGA TTT GAT CGC
pMAL-c5x-Pro45-63	TGA GAA TTC CCT GCA GGT	AAT CTG AGA TTT GAT CGC G
pMAL-c5x-Pro45-62	TGA GAA TTC CCT GCA GGT	CTG AGA TTT GAT CGC GTT TAG
pMAL-c5x-Pro45-61	TGA GAA TTC CCT GCA GGT	AGA TTT GAT CGC GTT TAG TC
pMAL-c5x-Pro45-60	TGA GAA TTC CCT GCA GGT	TTT GAT CGC GTT TAG TCG
pMAL-c5x-Pro46-70	GAC GTC CGG CAG CAG ATA	GAA TTC GGA TCC GTC GAC G
pMAL-c5x-Pro46-69	GAC GTC CGG CAG CAG ATA	GAA TTC GGA TCC GTC GAC G
pMAL-c5x-Pro47-70	GTC CGG CAG CAG ATA AAA	GAA TTC GGA TCC GTC GAC
pMAL-c5x-Pro49-70	CAG CAG ATA AAA ACC ATG C	GAA TTC GGA TCC GTC GAC
pMAL-c5x-Pro45-70-His6	CAC CAC CAC TGA GAA TTC CCT GCA GGT	ATG ATG ATG CTT CAT TCG CAG TTT GCT C
