# Supplementary figures and images for: Identification of the minimum region of flatfish myostatin propeptide (Pep45-65) for myostatin inhibition and its potential to enhance muscle growth and performance in animals
Source: PLoS One. 2019 Apr 18;14(4):e0215298. doi: 10.1371/journal.pone.0215298 (PMC6472743; doi:10.1371/journal.pone.0215298)

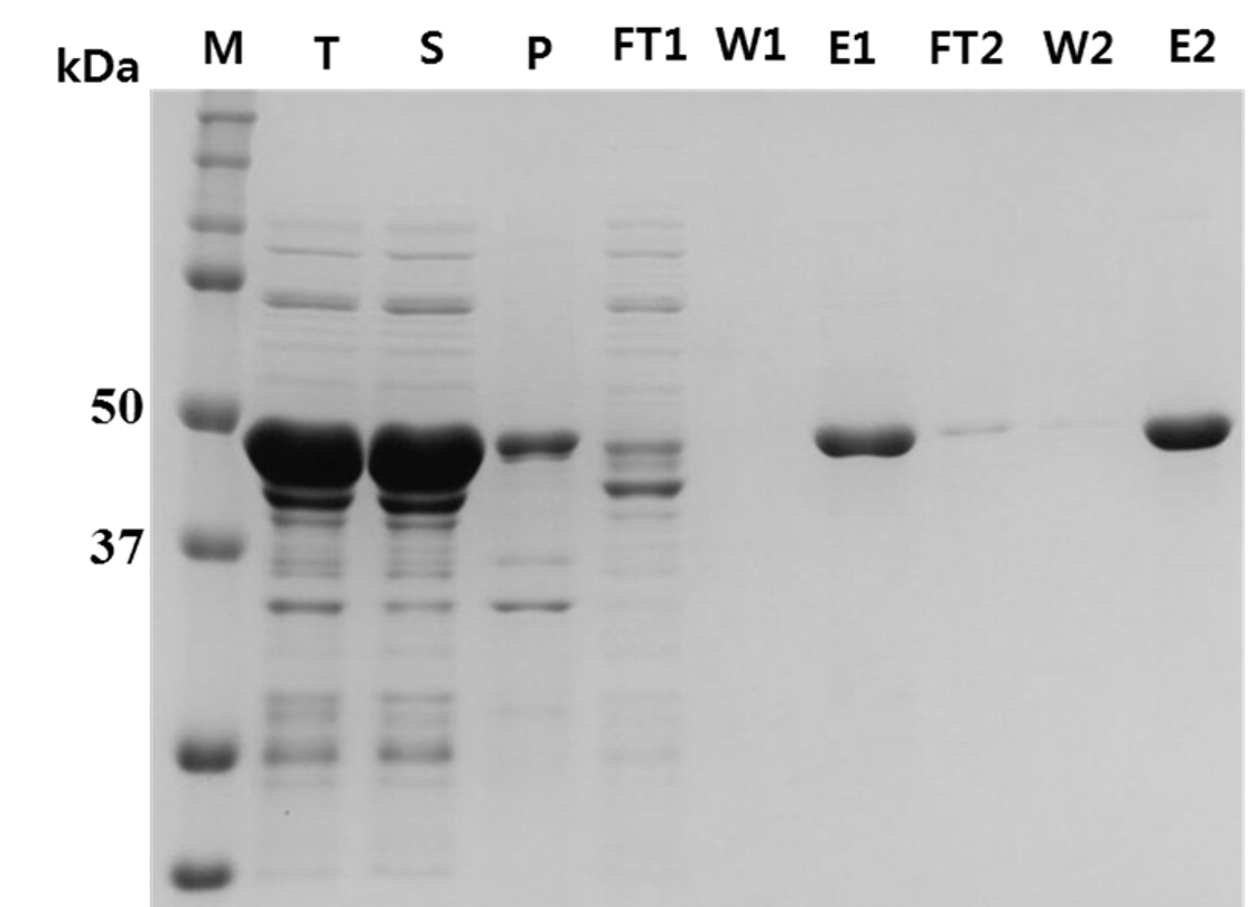

Supplement: S1 Fig — Ni-NTA agarose affinity chromatography was first used, followed by amylose resin affinity chromatography for purification. Proteins were visualized with Coomassie blue staining. M, protein ladder; T, total protein; S, soluble fraction; P: pellet (insoluble fraction), FT1: flow-through fraction of Ni-NTA agarose, W1: washing fraction of Ni-NTA agarose, E1: elution fraction of Ni-NTA agarose, FT2: flow-through fraction of amylose resin, W2: washing fraction of amylose resin, E2: elution fraction of amylose resin. (TIF) [file pone.0215298.s002.tif]

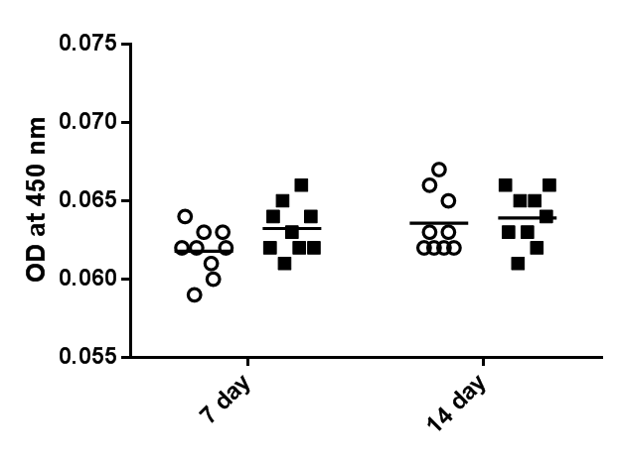

Supplement: S2 Fig — 1000-fold diluted sera were used for titer measurement. Open circle (○) and closed square (■) indicate 0 and 20 mg/kg body wt administration of Pro45-65-NH2, respectively. Student`s t-test was used to compare the mean difference. (TIF) [file pone.0215298.s003.tif]
